# Involvement of Neuropeptide Galanin Receptors 2 and 3 in Learning, Memory and Anxiety in Aging Mice

**DOI:** 10.3390/molecules26071978

**Published:** 2021-04-01

**Authors:** Felix Locker, Lara Bieler, Lioba M. F. Nowack, Julia Leitner, Susanne Maria Brunner, Pia Zaunmair, Barbara Kofler, Sebastien Couillard-Despres

**Affiliations:** 1 Research Program for Receptor Biochemistry and Tumor Metabolism, Department of Pediatrics, Paracelsus Medical University, Müllner Hauptstraße 48, 5020 Salzburg, Austria; Felix.Locker@vetmeduni.ac.at (F.L.); stocki93@gmx.at (J.L.); su.brunner@salk.at (S.M.B.); 2 Department of Biomedical Sciences, Institute of Physiology, Pathophysiology and Biophysics, University of Veterinary Medicine Vienna, 1210 Vienna, Austria; 3 Institute of Experimental Neuroregeneration, Spinal Cord Injury and Tissue Regeneration Center Salzburg (SCI-TReCS), Paracelsus Medical University, 5020 Salzburg, Austria; lara.bieler@pmu.ac.at (L.B.); lioba.nowack@stud.pmu.ac.at (L.M.F.N.); pia.zaunmair@gmail.com (P.Z.); s.couillard-despres@pmu.ac.at (S.C.-D.); 4 Austrian Cluster of Tissue Regeneration, 1010 Vienna, Austria

**Keywords:** learning, aging, galanin, neuropeptide, knock-out, behavior

## Abstract

The neuropeptide galanin (GAL), which is expressed in limbic brain structures, has a strong impact on the regulation of mood and behavior. GAL exerts its effects via three G protein-coupled receptors (GAL_1–3_-R). Little is known about the effects of aging and loss of GAL-Rs on hippocampal-mediated processes connected to neurogenesis, such as learning, memory recall and anxiety, and cell proliferation and survival in the dorsal dentate gyrus (dDG) in mice. Our results demonstrate that loss of *GAL_3_*-R, but not *GAL_2_*-R, slowed learning and induced anxiety in older (12–14-month-old) mice. Lack of *GAL_2_*-R increased cell survival (BrdU incorporation) in the dDG of young mice. However, normal neurogenesis was observed in vitro using neural stem and precursor cells obtained from *GAL_2_*-R and *GAL_3_*-R knockouts upon GAL treatment. Interestingly, we found sub-strain differences between C57BL/6J and C57BL/6N mice, the latter showing faster learning, less anxiety and lower cell survival in the dDG. We conclude that GAL-R signaling is involved in cognitive functions and can modulate the survival of cells in the neurogenic niche, which might lead to new therapeutic applications. Furthermore, we observed that the mouse sub-strain had a profound impact on the behavioral parameters analyzed and should therefore be carefully considered in future studies.

## 1. Introduction

In mice and humans, adult neurogenesis takes place in the sub-granular zone of the dorsal dentate gyrus (dDG) and the subventricular zone of the lateral ventricles [1,2,3]. Neurogenic events such as neural stem and progenitor cell proliferation, survival and integration are orchestrated by the neurogenic niche. The connection between learning and neurogenesis is complex and a matter of ongoing debate. Early work showed, for example, that cognitive tasks stimulate the generation of new neurons [4], whereas disruption of neurogenesis leads to cognitive impairment, which is also observed in aging [5]. Neuropeptides such as galanin (GAL) regulate various crucial functions of the central nervous system (CNS). However, direct involvement of GAL signaling in neurogenesis and learning, especially during aging, has not been investigated.

GAL is widely distributed in the central and peripheral nervous systems and regulates a variety of biological and physiological functions, ranging from energy homeostasis, reproduction and feeding to cognition and learning [6,7]. The GAL peptide is highly conserved throughout evolution and found in many species. GAL is processed from a 123 amino acid (aa) pre-pro-peptide, which gives rise to the mature GAL (29 aa in rodents, 30 aa in humans) and the GAL-message-associated peptide. GAL co-localizes with noradrenaline in the locus coeruleus of rats [8,9,10] and humans [11,12,13]. Additionally, GAL co-localizes with 5-hydroxytryptamine (serotonin) in the dorsal raphe nucleus of rats [9,14]. In mice, GAL mRNA is expressed at high levels in the amygdala, hypothalamus, locus coeruleus and the dentate gyrus of the hippocampus [15], which are crucial structures for the regulation of anxiety and stress [16,17]. GAL inhibits functions mediated by norepinephrine, serotonin, dopamine, glutamate and acetylcholine, which could be relevant for the pathophysiology of psychiatric disorders [18,19].

Three G-protein-coupled receptors (GPCRs) have been identified for GAL, namely GAL_1_-R, GAL_2_-R and GAL_3_-R. The GAL-Rs differ substantially in their functional coupling and signaling activities, thereby contributing to the diverse effects attributed to GAL [7]. GAL_1_-R mRNA is expressed in the mammalian CNS. In rodents, GAL_1_-R expression is especially high in the olfactory regions, subregions of the amygdala, hypothalamus, thalamus, medulla and spinal cord [20,21,22]. Expression of both GAL_1_-R and GAL_2_-R has been detected in the subventricular zone and the rostral migratory stream [23,24]. GAL_2_-R is found in the forebrain, midbrain and hindbrain, and it is highly expressed in the DG and hypothalamus. In the hindbrain, GAL_2_-R mRNA is located in the spinal trigeminal tract and the dorsal vagal complex as well as the paraventricular, ventromedial and dorsomedial nuclei of the hypothalamus [20,21,25]. Moderate expression of GAL_2_-R was found in large alpha motor neurons located in the ventral horn of the rat spinal cord. Spinal neurons in the gray matter, the ventral horns, in the dorsal horn and intermediate lateral cells (para- and sympathetic) were also found to express the GAL_2_-R [20,25]. GAL_3_-R expression in the CNS appears to be restricted to the hypothalamus [26,27,28].

Studies on various rodent models revealed an important role of GAL_3_-R in behavior. For example, Scheller and colleagues reported that administration of the GAL_3_-R antagonist SNAP 37,889 suppresses alcohol drinking and morphine self-administration in mice [29]. However, treatment of wild-type (WT) mice with GAL_3_-R antagonists such as SNAP 37,889 and SNAP 398,299 had an anxiolytic effect [30]. In contrast, Brunner and colleagues reported an anxiety-like phenotype in *GAL_3_*-knockout(KO) mice [31]. Furthermore, *GAL_2_*-KO mice displayed an anxiogenic-like phenotype [32].

The survival- and growth-promoting activities of GAL on different types of neurons in the peripheral and central nervous systems, including dorsal root ganglion (DRG) sensory neurons, have been well documented [33,34]. Several studies suggest that GAL_2_-R, and not GAL_1_-R, is primarily involved in the pro-regenerative activity of GAL on DRG and hippocampal neurons [35,36,37]. Because correlations between anxiety levels and neurogenesis have been suggested [38], and GAL-R signaling was shown to be involved in anxiety-related behaviors [31,32], we hypothesized that mice lacking *GAL2*-R or *GAL3*-R would behave differently from each other in anxiety-related tests and have different numbers of proliferating and newly integrated cells in the dDG.

In the present study, we analyzed neurogenesis-related behavioral and cognitive functions in young (3-month-old) and middle-aged (12–14-month-old) mice lacking *GAL2*-R or *GAL3*-R in comparison to the respective WT mice and quantified the numbers of proliferating and integrating cells in the dDG.

## 2. Results

### 2.1. Loss of GAL_3_-R Induces Anxiety in Middle-Aged Mice

**Figure 1:**
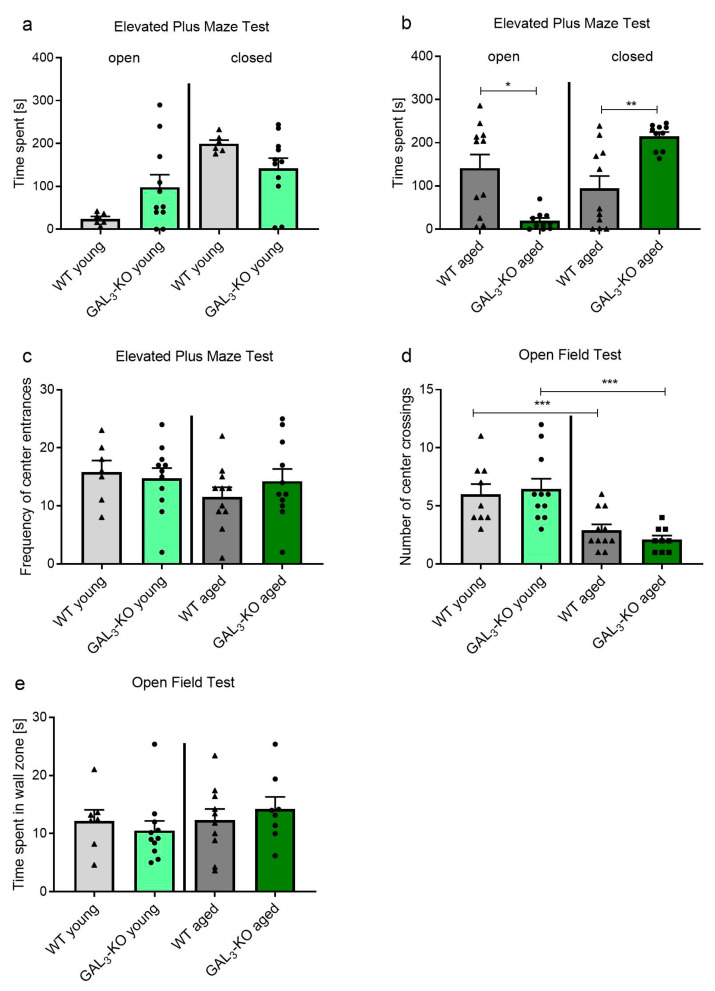
Behavior of *GAL_3_*-KOand WT mice in (**a**–**c**) EPM and (**d**,**e**) OF tests. Time spent in the open and closed arms of the EPM in (**a**) 3-month-old (young) and (**b**) 12–14-month-old (aged) mice. (**c**) Frequency of center entrances in the EPM in young and aged mice. (**d**) Number of center crossings and (**e**) time spent in the wall zone of the OF test in young and aged mice. Values are represented as mean ± SEM, n = 6–11 animals per group, * *p* < 0.05, ** *p* < 0.01, *** *p* < 0.001. *GAL_3_*-KO: *GAL_3_*-knockout; WT: Wildtype; EPM: Elevated Plus Maze; OF: Open Field.

We assessed the role of GAL-R in neurogenesis in relation to aging, which should correlate with anxiety-like behaviors in mice. The *GAL_2_*-KO and *GAL_3_*-KO mouse lines used in this study were bred on different genetic backgrounds, C57BL/6J and C57BL/6N, respectively. Therefore, statistical evaluation of differences of the KO-animals was restricted to the corresponding WT group unless otherwise stated. We tested ‘young-adult’ (3 months old) and ‘middle-aged’ (12–14 months old) *GAL_3_*-KO and WT mice for anxiety-related behavior using the elevated plus maze (EPM). In the EPM test, time spent in the closed arms of the apparatus, as opposed to the open arms or the central area, is considered an indicator of anxiety-related behavior. In the young cohort, EPM analysis revealed no significant difference in time spent in either the open or closed arms between *GAL_3_*-KO mice and WT controls (Figure 1a, time in open arms 24.2 ± 13.52 s (WT, n = 6) vs. 98.1 ± 96.2 s (*GAL_3_*-KO, n = 11), *p* = 0.08; time in closed arms: 199.4 ± 21.1 s (WT, n = 6) vs. 141.8 ± 80.3 s (*GAL_3_*-KO, n = 11), *p* = 0.11).

In contrast, for the older cohort, *GAL_3_*-KO mice stayed for significantly shorter lengths of time in the open arms and for significantly longer periods in the closed arms compared to the WT controls (Figure 1b, open arms: 141.5 ± 104.2 s (WT, n = 11) vs. 19.34 ± 21.74 s (*GAL_3_*-KO, n = 10), *p* = 0.002; closed arms: 94.8 ± 93.7 s (WT, n = 11) vs. 215.3 ± 29.9 s (*GAL_3_*-KO, n = 10), *p* = 0.001). Thus, older *GAL_3_*-KO mice presented significantly higher anxiety-like behavior compared to their age-matched WT counterparts. The frequency of center entrances (Figure 1c) did not differ between *GAL_3_*-KO and WT mice in their respective age groups (number of center entrances in the 3-month-old group: 15.9 ± 5.1 (WT, n = 7) vs. 14.7 ± 5.9 (*GAL_3_*-KO, n = 11), *p* = 0.683; 12–14-month-old group: 11.5 ± 5.5 (WT, n = 11) vs. 14.2 ± 6.9 (*GAL_3_*-KO, n = 11), *p* = 0.319).

In addition, we applied the open-field (OF) behavioral test because previous studies reported test-specific differences in behaviors for the GAL system [32,39]. The OF test provides an indication of spontaneous locomotory and exploratory behaviors. No significant differences in the number of center crossings were found between *GAL_3_*-KO and WT mice within their respective age groups (number of center crossings 3-month-old group: 6.0 ± 2.6 (WT, n = 11) vs. 6.4 ± 2.9 (*GAL_3_*-KO, n = 11), *p* = 0.723; 12–14-month-old group: 2.0 ± 1.7 (WT, n = 11) vs. 2.1 ± 1.0 (*GAL_3_*-KO, n = 9), *p* = 0.236, Figure 1d). However, the number of center crossings was significantly lower in both genotypes of older mice in comparison to young mice of the same genotype (6.0 ± 2.6 (3-month-old WT, n = 11) vs. 2.0 ± 1.7 (12–14-month-old WT, n = 11), *p* = 0.005; 6.4 ± 2.9 (3-month-old *GAL_3_*-KO, n = 11) vs. 2.1 ± 1.0 (12–14-month-old *GAL_3_*-KO, n = 9), *p* = 0.001). The time spent within the wall zone was similar between 3-month-old *GAL_3_*-KO mice and their age-matched WT counterparts (12.17 ± 5.1 s (WT, n = 7) vs. 10.5 ± 5.5 s (*GAL_3_*-KO, n = 11), *p* = 0.536) and between older *GAL_3_*-KO and WT mice (12.3 ± 6.0 s (WT, n = 10) vs. 14.2 ± 5.8 s (*GAL_3_*-KO, n = 8), *p* = 0.517, Figure 1e).

**Figure 2:**
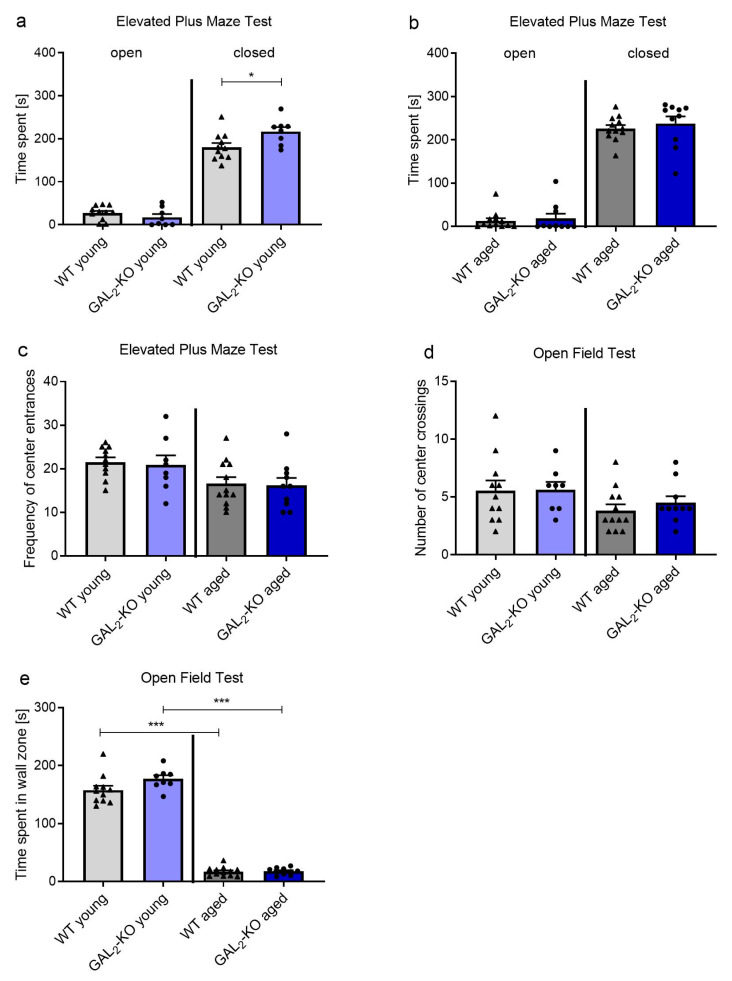
Behavior of *GAL_2_*-KO and WT mice in (**a**–**c**) EPM and (**d**,**e**) OF tests. Time spent in the open and closed arms of the EPM in (**a**) 3-month-old (young) and (**b**) 12–14-month-old mice (aged). (**c**) Frequency of center entrances in the EPM in young and aged mice. (**d**) Number of center crossings and (**e**) time spent in the wall zone of the OF test in young and aged mice. Values are represented as mean ± SEM, n = 8–12 animals per group, * *p* < 0.05, *** *p* < 0.001. *GAL_2_*-KO: *GAL_2_*-knockout; WT: Wildtype; EPM: Elevated Plus Maze; OF: Open Field.

We extended the same analyses to *GAL_2_*-KO mice. In the EPM, young *GAL_2_*-KO mice stayed longer in the closed arms compared to their age-matched WT controls, but no differences for the open arms were detected (closed arms: 180.5 ± 31.6 s (WT, n = 11) vs. 216.7 ± 30.0 s (*GAL_2_*-KO, n = 8), *p* = 0.024; open arms: 27.1 ± 16.0 s (WT, n = 11) vs. 17.4 ± 20.9 s (*GAL_2_*-KO n = 8), *p* = 0.277, Figure 2a). In the older mouse cohorts, no differences were observed between *GAL_2_*-KO and age-matched WT controls (closed arms: 226.0 ± 28.8 s (WT, n = 12) vs. 237.7 ± 52.4 s (*GAL_2_*-KO, n = 10), *p* = 0.512; open arms: 13.1 ± 21.1 s (WT, n = 12) vs. 18.7 ± 34.2 s (*GAL_2_*-KO, n = 10), *p* = 0.643, Figure 2b). Similar to *GAL_3_*-KO mice, the number of center entrances was unaffected by the loss of GAL_2_-R in both age groups (number of center entrances 3-month-old group: 21.5 ± 3.5 (WT, n = 11) vs. 20.8 ± 6.2 (*GAL_2_*-KO, n = 8), *p* = 0.770; 12–14-month-old group: 16.5 ± 5.1 (WT, n = 12) vs. 16.2 ± 5.4 (*GAL_2_*-KO, n = 10), *p* = 0.868, Figure 2c).

In the OF test, locomotion was similar between *GAL_2_*-KO and age-matched WT mice, independent of age (number of center crossings 3-month-old group: 5.5 ± 1.8 (WT, n = 12) vs. 5.6 ± 1.9 (*GAL_2_*-KO, n = 8), *p* = 0.947; 12–14-month-old group: 3.8 ± 1.8 (WT, n = 12) vs. 4.5 ± 1.7 (*GAL_2_*-KO, n = 10), *p* = 0.402, Figure 2d). The time spent within the wall zone was similar between the 3-month-old *GAL_2_*-KO group and WT (157.8 ± 25.4 s (WT, n = 11) vs. 177 ± 18.0 s (*GAL_2_*-KO, n = 8), *p* = 0.085, Figure 2e), thereby substantiating the similarity of the anxiety-related behavior between these two groups. Similarly, no differences in time spent within the wall zone were observed between the 12–14-month-old *GAL_2_*-KO and WT groups (17.0 ± 8.0 s (WT, n = 12) vs. 17.8 ± 5.9 s (*GAL_2_*-KO, n = 10), *p* = 0.796, Figure 2e). Interestingly, we found sub-strain-specific behavior in the OF test. While older mice on the C57BL/6J background, independent of genotype, spent significantly less time in the wall zone compared to younger mice (Figure 2e), mice on the C57BL/6N background showed similar behavior in both age groups (Figure 1e) (157.8 ± 25.4 s (3-month-old WT, n = 11) vs. 17.0 ± 8.0 s (12–14-month-old WT, n = 12), *p* < 0.001; 177 ± 18.0 s (3-month-old *GAL_2_*-KO, n = 8) vs. 17.8 ± 5.9 s (12–14-month-old *GAL_2_*-KO, n = 10), *p* < 0.001, Figure 2e).

### 2.2. Age-Dependent Deficit in Spatial Learning in Mice Lacking GAL_3_-R

**Figure 3:**
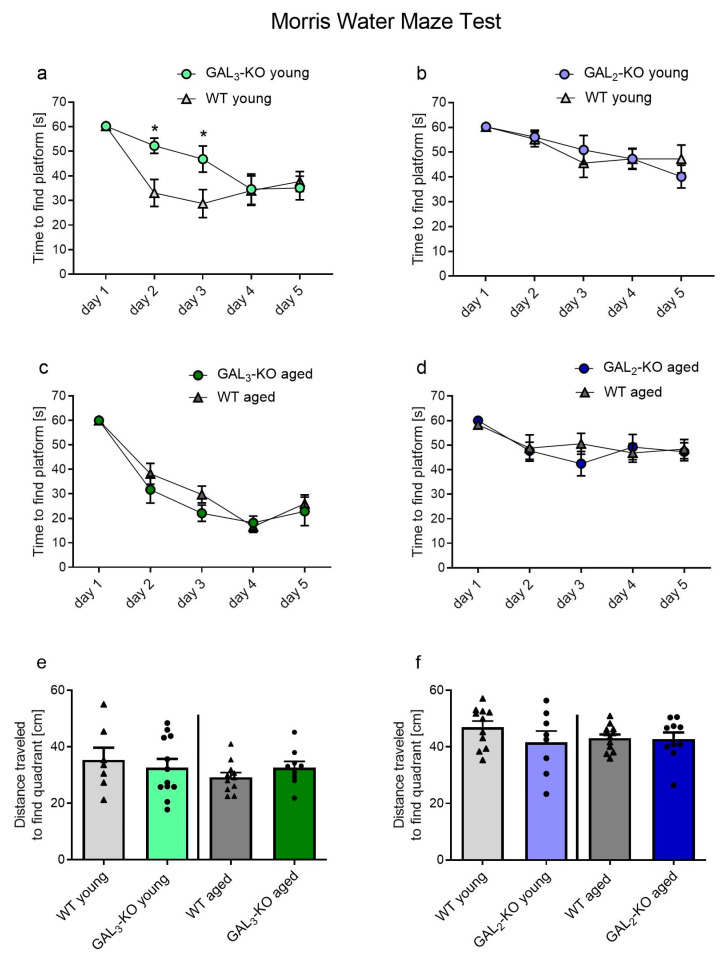
Behavior of *GAL_2_*-KO (**b**,**d**,**f**) and *GAL_3_*-KO (**a**,**c**,**e**) and the respective WT mice in the MWM test. Learning curves to find the platform in 3-month-old (young) (**a**,**b**) mice and 12–14-month-old (aged) (**c**,**d**) mice. (**e**,**f**) Memory assessment on day 6: required distance to travel to the quadrant where the platform was hidden on days 1–5 in (**e**) *GAL_3_*-KO and (**f**) *GAL_2_*-KO cohorts. Values are represented as mean ± SEM, n = 7–12 animals per group, * *p* < 0.05. *GAL_2_*-KO: *GAL_2_*-knockout; *GAL_3_*-KO: *GAL_3_*-knockout; WT: Wildtype; MWM: Morris Water Maze.

We assessed spatial learning with the Morris Water Maze (MWM) test, in which mice have to find and remember the position of a hidden platform on five consecutive days. Young *GAL_3_*-KO mice needed more time to find the platform than age-matched WT mice on day 2 and day 3 (day 2: *p* = 0.021, day 3: *p* = 0.034, Figure 3a). However, no differences were observed for the older *GAL_3_*-KO cohort compared to WT (Figure 3c) or the young or older *GAL_2_*-KO cohorts compared to WT (Figure 3b,d).

To assess memory, a single trial of 60 s was performed without the hidden platform 24 h after the last learning trial in the MWM test. The mean distance traveled to the platform’s location served as a parameter for memory. Mean distance to the platform did not differ between *GAL_3_*-KO mice and age-matched WT mice, independent of age (3-month-old group: 35.3 ± 11.3 cm (WT, n = 7) vs. 32.6 ± 10.4 cm (*GAL_3_*-KO, n = 12), *p* = 0.607; 12–14-month-old group: 29.2 ± 5.5 cm (WT, n = 11) vs. 32.6 ± 6.5 cm (*GAL_3_*-KO, n = 9), *p* = 0.217, Figure 3e). Similarly, *GAL_2_*-KO and controls did not differ in their memory-related behavior (3-month-old group: 47.0 ± 7.1 cm (WT, n = 11) vs. 41.6 ± 11.1 cm (*GAL_2_*-KO n = 8), *p* = 0.219; 12–14-month-old group: 43.1 ± 4.6 cm (WT, n = 12) vs. 42.8 ± 7.2 cm (*GAL_2_*-KO n = 10), *p* = 0.915, Figure 3f).

### 2.3. Deletion of GAL_2_-R Increases Survival of Newly Generated Cells in the dDG

**Figure 4:**
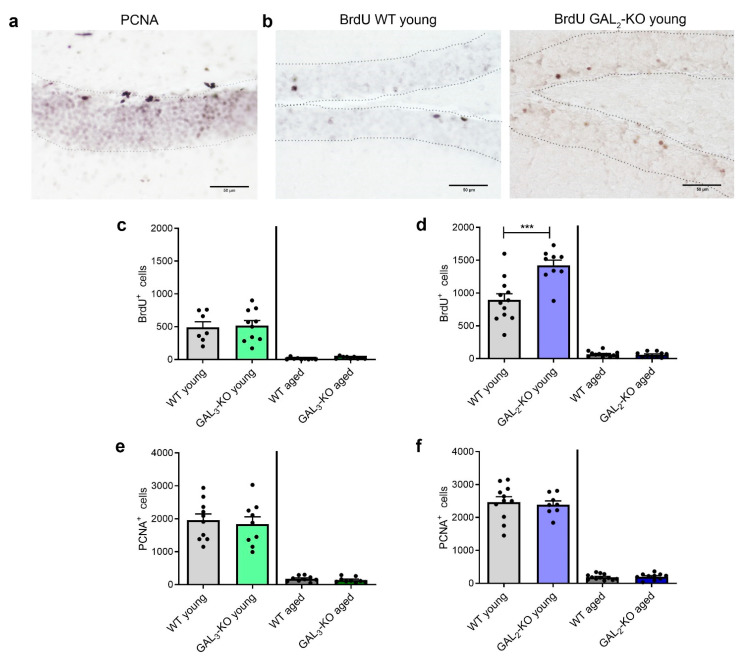
Immunohistochemical analyses of BrdU^+^ and PCNA^+^ cells in the DG. Representative micrographs of PCNA staining (**a**) in the DG of a 3-month-old mouse (C57BL/6N) and BrdU staining (**b**) of young WT and *GAL_2_*-KO mice (C57BL/6J). (**c**) Estimated numbers of BrdU^+^ cells in the DG of *GAL_3_*-KO and corresponding WT mice 28 days after the start of the experiment (WT: n = 10; other groups: n = 9). (**d**) Estimated numbers of BrdU^+^ cells in the DG of *GAL_2_*-KO and corresponding WT mice (WT young, WT aged: n = 12; *GAL_2_*-KO young: n = 9; *GAL_2_*-KO aged: n = 10). (**e**) Estimated numbers of PCNA^+^ cells in the DG of *GAL_3_*-KO and corresponding WT mice (WT: n = 10; other groups: n = 9). (**f**) Estimated numbers of PCNA^+^ cells in the dentate gyrus of *GAL_2_*-KO and corresponding WT mice (WT young: n = 11; WT aged: n = 12; *GAL_2_*-KO: n = 8; *GAL_2_*-KO aged: n = 10). Values are presented as mean ± SEM, *** *p* < 0.001; scale bar = 100 µm. BrdU: Bromodeoxyuridine; PCNA: proliferating cell nuclear antigen; *GAL_2_*-KO: *GAL_2_*-knockout; *GAL_3_*-KO: *GAL_3_*-knockout; WT: Wildtype.

To determine the degree of neurogenesis, we analyzed the number of integrated bromodeoxyuridine-labelled (BrdU^+^) as well as proliferating (proliferating-cell-nuclear-antigen: PCNA^+^) cells in the dDG of the mice after completing their behavioral trials. In the dDG of 3-month-old mice, similar amounts of BrdU^+^ cells were detected per hemisphere of *GAL_3_*-KO and WT mice (Figure 4c, 518.0 ± 242.0 (WT, n = 7) vs. 490.0 ± 229.0 (*GAL_3_*-KO, n = 10), *p* = 0.813). Due to the age-dependent reduction in neurogenesis, the 12–14-month-old mouse groups showed significantly less BrdU^+^ cells in comparison to young mice, but no genotype-specific differences were observed (Figure 4b,c, 11.1 ± 16.1 (WT, n = 9) vs. 26.6 ± 18.7 (*GAL_3_*-KO, n = 9), *p* = 0.077).

*GAL_2_*-KO mice showed higher levels of BrdU labelling in the 3-month-old group compared to their age-matched WT counterparts (Figure 4d, 893.3 ± 331.2 (WT, n = 12) vs. 1420.0 ± 249.5 (*GAL_2_*-KO, n = 9), *p* = 0.001). As expected, less BrdU^+^ cells were detected in the aged mice, but no genotype-associated difference was observed (65.0 ± 43.1 (WT, n = 12) vs. 60.0 ± 40.0 (*GAL_2_*-KO, n = 10), *p* = 0.782). Strikingly, the *GAL_2_*-KO and WT mouse cohorts, both on the C57BL/6J background, showed more than twice as many BrdU^+^ cells in the dDG compared to mice on the C57BL/6N background, independent of age (Figure 4c,d).

To discriminate the influence on proliferation from a change in survival of the newly generated cells in the dDG, the number of proliferating neural stem cells was estimated based on the presence of proliferating cell nuclear antigen (PCNA). Lack of *GAL_3_*-R did not change the number of PCNA^+^ cells in the dDG of the 3-month-old mice (Figure 4a,e, 1960.0 ± 599.9 (WT, n = 9) vs. 1841.1± 666.1 (*GAL_3_*-KO, n = 10), *p* = 0.686). Also, in the older mice, the loss of *GAL_3_*-R did not lead to a difference in the number of proliferating cells (Figure 4e, 175.6 ± 83.8 (WT, n = 9) vs. 141.1 ± 81.1 (*GAL_3_*-KO, n = 9), *p* = 0.388).

In the 3-month-old *GAL_2_*-KO mice and age-matched WT mice, no differences in the number of PCNA^+^ cells were measured in the dDG (Figure 4f, 2469.0 ± 539.8 (WT, n = 11) vs. 2393.0 ± 320.6 (*GAL_2_*-KO, n = 9), *p* = 0.725). Fewer PCNA^+^ cells were found in the older mouse brains of both WT and *GAL_2_*-KO mice (Figure 4f, 186.7 ± 86.06 (WT, n = 12) vs. 203.0 ± 95.5 (*GAL_2_*-KO, n = 10), *p* = 0.677). Hence, the increase in the number of BrdU^+^ cells observed in the 3-month-old *GAL_2_*-KO mice resulted from an improved survival of newly generated cells, rather than from a higher proliferation rate in the dDG.

### 2.4. Mouse Embryonic Forebrain Cells Express GAL System mRNA but Do Not Respond to GAL Treatment In Vitro

**Figure 5:**
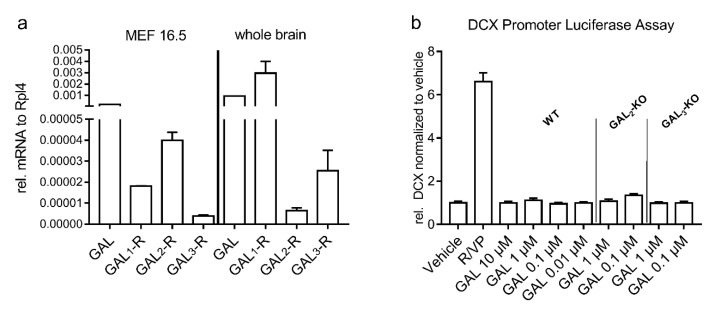
Gene expression analysis of the GAL-system. (**a**) Depicted are 2^−∆ct^ values from GAL and GAL_1–3_-R mRNA normalized to Rpl4 mRNA of mouse embryonic forebrain (MEF) cells from day 16.5 prenatal and C57BL/6N WT whole mouse brain. (**b**) Luciferase reporter activity driven by the DCX promoter in transfected primary MEF cells from day 16.5 prenatal. Cells were treated once with the peptides 3 days prior to the measurement. DCX Promoter experiments were performed at least in triplicates in 3 independent experiments, gene expression analysis was performed in duplicates and one representative experiment is shown. Values are represented as mean ± SEM. GAL, galanin; GAL_1_-R, galanin receptor 1; V/R, valproic/retinoic acid (positive control).

We detected expression of GAL and all three GAL-Rs in mouse embryonic forebrain (MEF) cultures derived from C57BL/6N WT mice (Figure 5a). Therefore, we analyzed the effect of signaling through the GAL-Rs on neurogenic processes in mouse E16.5 MEF cultures expressing the firefly luciferase reporter gene under the control of the doublecortin (DCX) promoter, an early neuronal marker [40,41]. Direct application of GAL in concentrations ranging from 0.01 to 10 µM did not induce DCX promoter activity in WT MEF cells (Figure 5b). We also treated MEF cells derived from *GAL_2_*-KO and *GAL_3_*-KO mice with GAL and did not observe an increase in the luciferase signal. Treatment of the various cultures with positive controls, i.e., the combination of valproic acid and retinoic acid, robustly increased the luciferase signal, thus confirming the potency of the cultured cells to undergo neuronal differentiation (Figure 5b).

### 2.5. C57BL/6N and C57BL/6J Sub-Strains Differ in Behavioral and Cognitive Tasks

**Figure 6:**
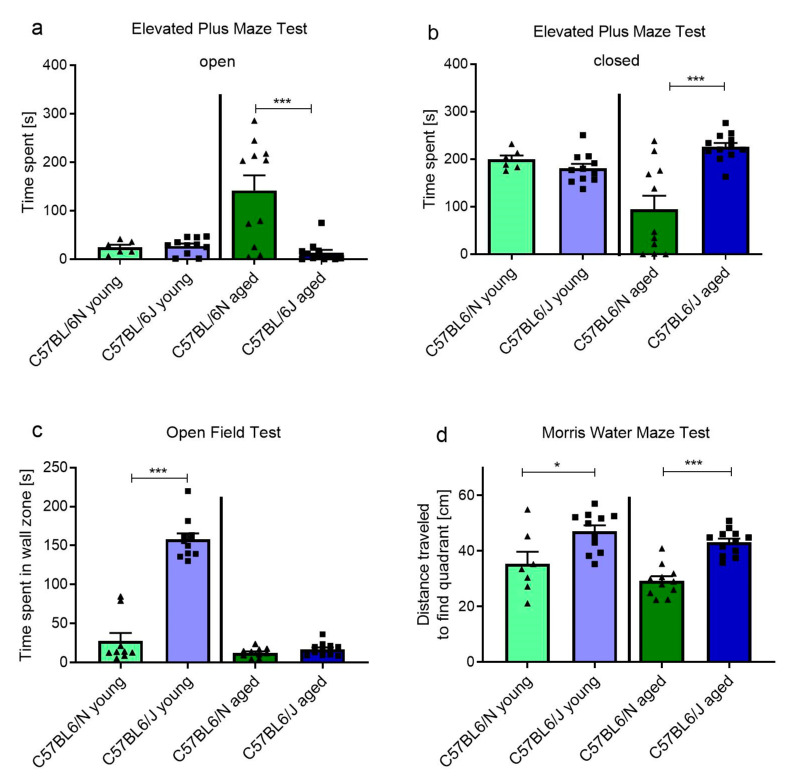
Behavior of C57BL/6N and C57BL/6J mice in (**a**,**b**) EPM, (**c**) OF and (**d**) Morris Water Maze (MWM) tests. Time spent in the (**a**) open and (**b**) closed arms of the EPM, (**c**) frequency of center entrances in the OF and (**d**) distance traveled to find the platform quadrant in the MWM. Values are represented as mean ± SEM, n = 6–11 animals per group, * *p* < 0.05, *** *p* < 0.001. OF: Open Field.

From the results of the various behavioral tests, we observed differences between WT mice on the C57BL/6J and C57BL/6N genetic backgrounds. We retrospectively compared the performances in the behavioral tests of the WT groups. The time spent in the open arm of the EPM did not differ significantly in young mice (27.1 ± 16.5 s (C57BL/6J, n = 11) vs. 24.2 ± 13.5 s (C57BL/6N, n = 6), Figure 6a). However, the two sub-strains of older mice differed significantly from each other (13.1 ± 21.1 s (C57BL/6J, n = 12) vs. 141.5 ± 104.2 s (C57BL/6N, n = 11), *p* < 0.001, Figure 6a). A similar anxiety-like behavior was observed in the OF test, where young C57BL/6J mice stayed longer in the wall zone compared to age-matched C57BL/6N mice (157.8 ± 25.4 s (C57BL/6J, n = 11) vs. 12.17 ± 5.1 s (C57BL/6N, n = 7), *p* < 0.001, Figure 6c). Interestingly, we also detected memory recall differences between C57BL/6J and C57BL/6N mice, independent of age. Young and older C57BL/6J mice traveled longer to find the quadrant where the platform was hidden compared to age-matched C57BL/6N mice (3-month-old mice: 47.3 ± 7.1 s (C57BL/6J, n = 11) vs. 35.3 ± 11.3 s (C57BL/6N, n = 7), *p* = 0.0165; 12–14-month-old mice: 43.1 ± 4.6 s (C57BL/6J, n = 12) vs. 29.2 ± 5.5 s (C57BL/6N, n = 11), *p* = 0.0054, Figure 6d). In addition, survival of neural precursors in the dDG (BrdU) was significantly lower in C57BL/6N compared to C57BL/6J, independent of age (3-month-old mice: 490 ± 229 (C57BL/6N, n = 7) vs. 893 ± 331 (C57BL/6J, n = 12), *p* = 0.0114; 12–14-month-old mice: 11 ± 16 (C57BL/6N, n = 9) vs. 65 ± 43 (C57BL/6J, n = 12), *p* = 0.0022, Figure 4c,d), whereas proliferation (PCNA) did not differ between the sub-strains (3-month-old mice: 1960 ± 599 (C57BL/6N, n = 10) vs. 2469 ± 539 (C57BL/6J, n = 11), *p* = 0.054; 12–14-month-old mice: 175 ± 83 (C57BL/6N, n = 9) vs. 186 ± 86 (C57BL/6J, n = 12) *p* = 0.7704, Figure 4e,f).

## 3. Discussion

In the present study, we submitted young and aged *GAL_2_*-KO and *GAL_3_*-KO mice to a spatial learning task and two anxiety-related behavioral tests to examine if the GAL system is involved in these cognitive functions and affected by aging. Furthermore, we scrutinized for potential correlations between behavior and the levels of cell proliferation and survival within the neurogenic niche of the hippocampus as a function of age. We observed that young *GAL_3_*-KO mice show reduced spatial learning in the MWM test compared to WT, whereas older *GAL_3_*-KO mice develop an anxiety-like behavior, according to the EPM test. This *GAL_3_*-KO anxiety-like phenotype is in line with previous findings from our laboratory, although the anxiety-like phenotype was also detected in young *GAL_3_*-KO mice on the C57BL/6N background and in the OF test in our previous study [31]. A possible explanation could be the occurrence of genetic drift in this mouse line in the time since the previous study, although the lines were backcrossed to the C57BL/6N background every seventh generation. Additionally, we confirmed genetic uniformity to the C57BL/6N genetic background by genetic homogeneity analysis performed by the mouse distributor (data not shown). The behavior of mice on the C57BL/6J background was similar, independent of age or *GAL_2_*-KO genotype (except for the EPM test, where young *GAL_2_*-KO mice spent more time in the closed arms). Previous findings from Bailey and colleagues reported a more robust anxiety-like behavioral response for *GAL_2_*-KO mice in the EPM. Importantly, their mice were on the 129S1Sv/ImJ genetic background [32]. Further background-specific phenotypes were observed in *GAL*-KO mice on the 129/OlaHsd background, which had a severe lactation deficit [32,33]. Intriguingly, Bailey et al. also reported EPM- but not OF-related anxiety-like behavior in *GAL_1_*-KO mice [32,39]. Holmes and colleagues gave a possible explanation for this anxiety-like behavior restricted to the EPM. They reported that the EPM test leads to especially high secretion of stress hormones as compared to other behavioral tests such as the OF. These circulating hormones, such as adrenocorticotropic hormone (ACTH) and corticosterone, may lead to a different stress-induced GAL response during the EPM as compared to other behavioral challenges [39]. Stress response circuits are highly conserved between different mammalian species and were shown to be already present in the earliest vertebrates [42]. Therefore, we speculate that, given the evolutionary importance of stress responses, genetic compensation still guarantees adequate responses in KO mice, as reported previously for other genes, where phenotypes in knockdowns and KO differed significantly [43]. Therefore, we hypothesize that the effects of a full germ-line KO of *GAL_2_*-R or *GAL_3_*-R might lead to partial compensation due to the latter mentioned genetic robustness of the system, which might decline during aging, resulting in the observed increased anxiety-like behavior of *GAL_3_*-KO mice.

Interestingly, the deficit in learning and the anxiety-like behavior detected in *GAL_3_*-KO mice were not reflected by changes in proliferation and survival of cells in the neurogenic niche of the dDG. In line with this, no change in the activity of the DCX promoter, which correlates with neurogenic activity, was observed following application of GAL to MEF cell cultures. Mennicken et al. [28] demonstrated that *GAL_3_*-R appears to be restricted to the hypothalamus. The hypothalamus is, beside the hippocampus, part of the limbic system, which is strongly associated with memory formation and emotion [44]. Therefore, we think that the therapeutic enhancement of *GAL_3_*-signaling could improve age-related comorbidities like loss of memory function or anxiety. However, a direct correlation to the neurogenic niche seems to be unlikely. In contrast, we detected improved survival of newly generated cells in the dDG of 3-month-old *GAL_2_*-KO mice. Hence, no direct correlation was observed between the proliferation and survival of newly generated cells in the dDG of mice with *GAL_2_*-R or *GAL_3_*-R KO and their performance in the behavioral tests. However, in accordance with the literature [5], we observed drastic reductions of proliferating and integrating cells in the older mice of both lines. Interestingly, we detected differences in learning capabilities and behavior between C57BL/6N and C57BL/6J mice which adds knowledge to previously reported differences between C57BL/6N and C57BL/6J mice ranging from chronobiology and metabolic responses [45] to general behavior [46,47]. We speculate that the lower learning capabilities of C57BL/6J mice and their overall anxious behavior upon aging compared to C57BL/6N mice might result from the overall increased proliferation of cells in the dDG, which was shown to induce “forgetting” of unpleasant events [48].

## 4. Material and Methods

### 4.1. Animals

Mice were bred and housed in the animal facility of the Paracelsus Medical University. *GAL_3_*-KO mice were initially obtained from the European Mouse Mutant Archive and were backcrossed to the C57BL/6N background and phenotypically characterized in our lab [31]. *GAL_2_*-KO on the C57BL/6J background [49] were kindly provided by Marina Picciotto’s lab (Yale University). KO and WT mice were bred as homozygotes. C57BL/6N and C57BL/6J mice (Charles River, Sulzfeld, Germany) were used to backcross *GAL_2_*-KO and *GAL_3_*-KO mice every 7th generation to limit genetic drift. All animals were analyzed with respect to their GAL_2_-R and GAL_3_-R genotype before and after the experiments [31,49]. Male mice were used exclusively in the study to avoid effects of the estrous cycle on the results. All mice were kept in groups of a maximum of five, with a 12 h light/dark cycle and access to water and food ad libitum. All experiments were approved by the Austrian federal ethical commission (BMBWF-66.019/0031-V/3b/2018) and were conducted in accordance with the guidelines of “Directive 2010/63/EU of the European Parliament and of the Council of 22 September 2010 on the protection of animals used for scientific purposes”.

### 4.2. BrdU Injection

To study survival and integration of newly generated cells in the dDG, the DNA of dividing cells was labeled through daily intraperitoneal injections of 50 mg/kg bodyweight BrdU (Sigma-Aldrich, Inc., Darmstadt, Germany) from day 1 to 4. Following perfusion on day 28, BrdU^+^ cells were visualized by immunohistochemical techniques.

### 4.3. Elevated Plus Maze

Elevated Plus Maze (EPM) (Ugo Basile, Gemonio, Italy) trials were performed on day 19 after the start of the BrdU injections, as previously described [50]. Mice were placed in the central region of the maze and their position was tracked for 5 min using an Ethovision XT system (Noldus, Wageningen, The Netherlands). The maze was cleaned between every trial to eliminate olfactory cues. The time spent in the open arms and central region was designated as exploratory behavior, whereas time spent in the closed arms was defined as anxious-like behavior. For statistical analyses, total time spent in the open/center zone vs. total time spent in closed arms was analyzed.

### 4.4. Open Field

The open-field (OF) test, which assesses spontaneous locomotor and exploratory activities, was performed on day 20 after the start of the BrdU injections. Mice were placed in the middle of a circular arena 1 m in diameter and allowed to move freely for 5 min. Parameters of movement and position were recorded using Ethovision XT software (Noldus, Wageningen, The Netherlands). The arena was cleaned between every trial to eliminate olfactory cues.

### 4.5. Morris Water Maze

The Morris Water Maze (MWM) test was performed to assess spatial learning and memory from day 23 to 28 after the start of the BrdU injections, as previously described [50]. The time taken to find the hidden platform was recorded using Ethovision XT tracking software (Noldus, Wageningen, The Netherlands). For the evaluation of memory on day 28, the trial was performed in the absence of the hidden platform. Time spent in the platform quadrant, as well as mean distance to the platform and numbers of platform crossings were analyzed.

### 4.6. Immunohistochemistry

Mice were transcardially perfused with 0.9% NaCl for 7 min, followed by 0.1 M phosphate-buffered 4% paraformaldehyde (pH 7.4) for 7 min. Brains were dissected and post-fixed in the same solution overnight at 4 °C and transferred to 0.1 M phosphate-buffered 30% sucrose solution (pH 7.4) at 4 °C for at least 48 h. Brains were cut into 40 μm sagittal sections using a sliding microtome (Leica, Vienna, Austria) on dry ice. Sections were stored at −20 °C in cryo-protectant solution (25% *v*/*v* glycerol, 0.05 M sodium phosphate buffer, pH 7.4, 25% *v*/*v* ethylene glycol) until further processing. Immunohistochemical analyses were performed as previously described [51] using the following antibodies and kits: rat anti-BrdU (AbD Serotec, 1:500), mouse anti-PCNA (Santa Cruz Biotechnology, Heidelberg, Germany, 1:500), biotinylated rabbit anti-rat antibody (Vector Laboratories, Szabo-Scandic, Vienna, Austria, 1:1000), biotinylated donkey anti-mouse antibody (Jackson Immuno Research, Szabo-Scandic, Vienna, Austria, 1:1000), VECTASTAIN ABC System and DAB Peroxidase Substrate kit (Vector Laboratories Szabo-Scandic, Vienna, Austria) and 4′6-diamidino-2-phenylindole (DAPI 0.5 μg/mL, Sigma-Aldrich, Darmstadt, Germany). The total number of stained cells per hemisphere in the granular and sub-granular layers of the dDG was extrapolated using stereological techniques on every tenth section, as described previously [51]. Positive-stained cells were counted manually at 40× magnification using an upright Nikon Eclipse 600 microscope (Amsterdam, The Netherlands). Bright-field micrographs were acquired with a vs. 120 Slide Scanner (Olympus, Hamburg, Germany).

### 4.7. Mouse Embryonic (E16.5) Forebrain Cell Culture

Mouse embryonic forebrain (MEF) cells were isolated from E16.5 mouse embryos from WT, *GAL_2_*-KO and *GAL_3_*-KO mice, and processed as previously described [52]. On the fourth day after harvesting, cells were transiently co-transfected using an Amaxa Nucleofection Kit (Program A033; Lonza, Basel, Switzerland) with a plasmid encoding the human doublecortin (DCX) promoter driving the firefly luciferase gene. The human DCX promoter is induced early upon neuronal differentiation and serves as a reliable marker for the latter [40,41]. Afterwards, transfected cells were seeded in 100 µg/mL p-L-ornithine and 5 µg/mL laminin-coated white 96-well plates (25,000 cells/well). After 24 h, cells were treated with 10 µM, 1 µM and 100 nM GAL peptide. Treatment with 50 µM valproic acid (Sigma, Darmstadt, Germany) in combination with 10 µM retinoic acid (Sigma, Darmstadt, Germany) served as a positive control, whereas medium with vehicle without GAL peptide (endotoxin-free, sterile water with 0.1% bovine serum albumin (BSA, Sigma, Darmstadt, Germany) served as a negative control. The cells were further incubated for 3 days with the compounds to allow neuronal differentiation, prior to cell lysis and final measurement of luciferase activity using a multi-well reader (Berthold, Bad Wildbad, Germany), as described previously [52]. The luciferase activity of cells treated with the peptide and that of the positive control were normalized to the luciferase activity of the vehicle-treated controls.

### 4.8. RNA Analysis

RNA of MEF cells (E 16.5) and whole brain of C57BL/6N mice were isolated using TRI-Reagent (Molecular Research Center, Cincinnati, OH, USA) following the manufacturer’s protocol, as described previously [53]. Maxima Reverse Transcriptase (Thermo Scientific, Pittsburgh, PA, USA) and random hexamer primers were used for cDNA synthesis according to the manufacturer’s instructions. To quantify GAL and GAL-R expression, iQ SYBR green super-mix (BioRad, Vienna, Austria) was used. Forward and reverse primers were synthesized by Microsynth (Balgach, Switzerland), with the respective target sites separated by at least one intron (GAL: fwd 5′-ACCAGGAAGTGTTGATGTGCC-3′, rev 5′-TCTAGGTCTTCTGAGGAGGTGG-3′; GAL_1_-R: fwd 5′-CTGCCCTTACTGCTCATCTGC-3′, rev 5′-TACAACGACCACCAGGACGG-3′; GAL_2_-R fwd 5′-TCTCGCTGGACAGGTATCTGG-3′, rev 5′-CTGACTGTAGTAGCTCAGGTAGGG-3′; GAL_3_-R: fwd 5′-GGCCGTCTCAGTGGATAGGT-3′, rev 5′-AGCTTAGGTAGGGCGCGGA-3′; Ribosomal protein L 4 (mRPL4): fwd 5′-GTATGGCACTTGGCGGAAGG-3′, rev 5′-TGCTCGGAGGGCTCTTTGG-3′). The amplification reaction was performed in duplicates for 40 cycles (97 °C for 15 s, 63 °C for 30 s and 72 °C for 10 s). The relative expression of the genes was determined by the delta threshold cycle (Ct) (2^-(Ct of the gene of interest − Ct of the housekeeping gene RPL4)).

### 4.9. Peptide

Rat-galanin 1-29 amide (GL Biochem, Shanghai, China) was diluted in Hank’s balanced salt solution (HBSS) without magnesium and calcium (HBSS -/-) (Sigma-Aldrich, Darmstadt, Germany) to the respective concentrations. The peptide stock solution consisted of 0.1% BSA in endotoxin-free and sterile Aqua Ampuwa (Fresnius Kabi, Bad Homburg, Germany). The stock peptide and diluted peptide were stored and diluted in Protein LoBind tubes (Eppendorf, Hamburg, Germany).

### 4.10. Statistical Analysis

All data were analyzed using GraphPad Prism 7 (Graphpad Inc., San Diego, CA, USA). Unpaired Student’s *t*-test and one-way analysis of variance (ANOVA) were used to evaluate differences between mice. Outliers were identified using the ROUT test [54] and were excluded for further analyses. A *p*-value ≤ 0.05 was considered significant. All values are represented as mean ± standard error of the mean (SEM).

## 5. Conclusions

We showed that neuropeptide GAL signaling through GAL_3_-R is relevant for learning and anxiety in an age-dependent manner. In addition, we observed that proliferation of cells in the neurogenic niche can be modulated by GAL signaling; however, there was no correlation with the behavioral outcome. Furthermore, we want to emphasize that the genetic background of the animal models used can significantly impact behavioral outcomes, which underscores the high complexity of analyzing neuropeptide functions in mice. Finally, we conclude that the GAL neuropeptide system might be a promising target for the development of therapeutics which target age-related comorbidities such as anxiety or reduced memory function either as an antagonist (GAL_2_-R) or agonist (GAL_3_-R). However, a direct correlation of *GAL_3_*-signaling and the neurogenic niche seems to be unlikely.

## Data Availability

The data presented in this study are available on request from the corresponding author.

## Sample Availability

Remaining tissue samples of the study are available from the authors.

## References

[1] Kuhn H.G., Dickinson-Anson H., Gage F.H. (1996). Neurogenesis in the dentate gyrus of the adult rat: Age-related decrease of neuronal progenitor proliferation. J. Neurosci..

[2] Luskin M.B. (1993). Restricted proliferation and migration of postnatally generated neurons derived from the forebrain subventricular zone. Neuron.

[3] Kempermann G., Gage F.H., Aigner L., Song H., Curtis M.A., Thuret S., Kuhn H.G., Jessberger S., Frankland P.W., Cameron H.A. (2018). Human adult neurogenesis: Evidence and remaining questions. Cell Stem Cell.

[4] Gould E., Tanapat P., Hastings N.B., Shors T.J. (1999). Neurogenesis in adulthood: A possible role in learning. Trends Cogn. Sci..

[5] Toda T., Parylak S.L., Linker S.B., Gage F.H. (2019). The role of adult hippocampal neurogenesis in brain health and disease. Mol. Psychiatry.

[6] Lang R., Gundlach A.L., Kofler B. (2007). The galanin peptide family: Receptor pharmacology, pleiotropic biological actions, and implications in health and disease. Pharmacol. Ther..

[7] Lang R., Gundlach A.L., Holmes F.E., Hobson S.A., Wynick D., Hokfelt T., Kofler B. (2015). Physiology, signaling, and pharmacology of galanin peptides and receptors: Three decades of emerging diversity. Pharmacol. Rev..

[8] Holets V.R., Hokfelt T., Rokaeus A., Terenius L., Goldstein M. (1988). Locus coeruleus neurons in the rat containing neuropeptide y, tyrosine hydroxylase or galanin and their efferent projections to the spinal cord, cerebral cortex and hypothalamus. Neuroscience.

[9] Melander T., Hokfelt T., Rokaeus A., Cuello A.C., Oertel W.H., Verhofstad A., Goldstein M. (1986). Coexistence of galanin-like immunoreactivity with catecholamines, 5-hydroxytryptamine, gaba and neuropeptides in the rat cns. J. Neurosci..

[10] Hokfelt T., Xu Z.Q., Shi T.J., Holmberg K., Zhang X. (1998). Galanin in ascending systems. Focus on coexistence with 5-hydroxytryptamine and noradrenaline. Ann. N. Y. Acad. Sci..

[11] Kordower J.H., Le H.K., Mufson E.J. (1992). Galanin immunoreactivity in the primate central nervous system. J. Comp. Neurol..

[12] Miller M.A., Kolb P.E., Leverenz J.B., Peskind E.R., Raskind M.A. (1999). Preservation of noradrenergic neurons in the locus ceruleus that coexpress galanin mrna in alzheimer’s disease. J. Neurochem..

[13] Le Maitre E., Barde S.S., Palkovits M., Diaz-Heijtz R., Hokfelt T.G. (2013). Distinct features of neurotransmitter systems in the human brain with focus on the galanin system in locus coeruleus and dorsal raphe. Proc. Natl. Acad. Sci. USA.

[14] Lu X., Barr A.M., Kinney J.W., Sanna P., Conti B., Behrens M.M., Bartfai T. (2005). A role for galanin in antidepressant actions with a focus on the dorsal raphe nucleus. Proc. Natl. Acad. Sci. USA.

[15] Cheung C.C., Hohmann J.G., Clifton D.K., Steiner R.A. (2001). Distribution of galanin messenger rna-expressing cells in murine brain and their regulation by leptin in regions of the hypothalamus. Neuroscience.

[16] McCall J.G., Al-Hasani R., Siuda E.R., Hong D.Y., Norris A.J., Ford C.P., Bruchas M.R. (2015). Crh engagement of the locus coeruleus noradrenergic system mediates stress-induced anxiety. Neuron.

[17] Davis M. (1992). The role of the amygdala in fear and anxiety. Annu. Rev. Neurosci..

[18] Karlsson R.M., Holmes A. (2006). Galanin as a modulator of anxiety and depression and a therapeutic target for affective disease. Amino Acids.

[19] Blier P., El Mansari M. (2007). The importance of serotonin and noradrenaline in anxiety. Int. J. Psychiatry Clin. Pract..

[20] O’Donnell D., Ahmad S., Wahlestedt C., Walker P. (1999). Expression of the novel galanin receptor subtype galr2 in the adult rat cns: Distinct distribution from galr1. J. Comp. Neurol..

[21] Burazin T.C., Larm J.A., Ryan M.C., Gundlach A.L. (2000). Galanin-r1 and -r2 receptor mrna expression during the development of rat brain suggests differential subtype involvement in synaptic transmission and plasticity. Eur. J. Neurosci..

[22] Hohmann J.G., Jureus A., Teklemichael D.N., Matsumoto A.M., Clifton D.K., Steiner R.A. (2003). Distribution and regulation of galanin receptor 1 messenger rna in the forebrain of wild type and galanin-transgenic mice. Neuroscience.

[23] Shen P.J., Larm J.A., Gundlach A.L. (2003). Expression and plasticity of galanin systems in cortical neurons, oligodendrocyte progenitors and proliferative zones in normal brain and after spreading depression. Eur. J. Neurosci..

[24] Mazarati A.M. (2004). Galanin and galanin receptors in epilepsy. Neuropeptides.

[25] Gundlach A.L., Burazin T.C. (1998). Galanin-galanin receptor systems in the hypothalamic paraventricular and supraoptic nuclei. Some recent findings and future challenges. Ann. N. Y. Acad. Sci..

[26] Brumovsky P., Mennicken F., O’Donnell D., Hokfelt T. (2006). Differential distribution and regulation of galanin receptors-1 and -2 in the rat lumbar spinal cord. Brain Res..

[27] Hawes J.J., Picciotto M.R. (2004). Characterization of galr1, galr2, and galr3 immunoreactivity in catecholaminergic nuclei of the mouse brain. J. Comp. Neurol..

[28] He B., Counts S.E., Perez S.E., Hohmann J.G., Koprich J.B., Lipton J.W., Steiner R.A., Crawley J.N., Mufson E.J. (2005). Ectopic galanin expression and normal galanin receptor 2 and galanin receptor 3 mrna levels in the forebrain of galanin transgenic mice. Neuroscience.

[29] Mennicken F., Hoffert C., Pelletier M., Ahmad S., O’Donnell D. (2002). Restricted distribution of galanin receptor 3 (galr3) mrna in the adult rat central nervous system. J. Chem. Neuroanat..

[30] Scheller K.J., Williams S.J., Lawrence A.J., Djouma E. (2017). The galanin-3 receptor antagonist, snap 37889, suppresses alcohol drinking and morphine self-administration in mice. Neuropharmacology.

[31] Swanson C.J., Blackburn T.P., Zhang X., Zheng K., Xu Z.Q., Hokfelt T., Wolinsky T.D., Konkel M.J., Chen H., Zhong H. (2005). Anxiolytic- and antidepressant-like profiles of the galanin-3 receptor (gal3) antagonists snap 37889 and snap 398299. Proc. Natl. Acad. Sci. USA.

[32] Brunner S.M., Farzi A., Locker F., Holub B.S., Drexel M., Reichmann F., Lang A.A., Mayr J.A., Vilches J.J., Navarro X. (2014). Gal3 receptor ko mice exhibit an anxiety-like phenotype. Proc. Natl. Acad. Sci. USA.

[33] Bailey K.R., Pavlova M.N., Rohde A.D., Hohmann J.G., Crawley J.N. (2007). Galanin receptor subtype 2 (galr2) null mutant mice display an anxiogenic-like phenotype specific to the elevated plus-maze. Pharmacol. Biochem. Behav..

[34] Mahoney S.A., Hosking R., Wynick D. (2003). The galanin antagonist m35 has intrinsic agonistic activity in the dorsal root ganglion. Neuroreport.

[35] Holmes F.E., Mahoney S., King V.R., Bacon A., Kerr N.C., Pachnis V., Curtis R., Priestley J.V., Wynick D. (2000). Targeted disruption of the galanin gene reduces the number of sensory neurons and their regenerative capacity. Proc. Natl. Acad. Sci. USA.

[36] Mahoney S.A., Hosking R., Farrant S., Holmes F.E., Jacoby A.S., Shine J., Iismaa T.P., Scott M.K., Schmidt R., Wynick D. (2003). The second galanin receptor galr2 plays a key role in neurite outgrowth from adult sensory neurons. J. Neurosci..

[37] Elliott-Hunt C.R., Pope R.J., Vanderplank P., Wynick D. (2007). Activation of the galanin receptor 2 (galr2) protects the hippocampus from neuronal damage. J. Neurochem..

[38] Elliott-Hunt C.R., Marsh B., Bacon A., Pope R., Vanderplank P., Wynick D. (2004). Galanin acts as a neuroprotective factor to the hippocampus. Proc. Natl. Acad. Sci. USA.

[39] Hill A.S., Sahay A., Hen R. (2015). Increasing adult hippocampal neurogenesis is sufficient to reduce anxiety and depression-like behaviors. Neuropsychopharmacology.

[40] Holmes A., Kinney J.W., Wrenn C.C., Li Q., Yang R.J., Ma L., Vishwanath J., Saavedra M.C., Innerfield C.E., Jacoby A.S. (2003). Galanin gal-r1 receptor null mutant mice display increased anxiety-like behavior specific to the elevated plus-maze. Neuropsychopharmacology.

[41] Karl C., Couillard-Despres S., Prang P., Munding M., Kilb W., Brigadski T., Plotz S., Mages W., Luhmann H., Winkler J. (2005). Neuronal precursor-specific activity of a human doublecortin regulatory sequence. J. Neurochem..

[42] Couillard-Despres S., Quehl E., Altendorfer K., Karl C., Ploetz S., Bogdahn U., Winkler J., Aigner L. (2008). Human in vitro reporter model of neuronal development and early differentiation processes. BMC Neurosci..

[43] Denver R.J. (2009). Structural and functional evolution of vertebrate neuroendocrine stress systems. Ann. N. Y. Acad. Sci..

[44] El-Brolosy M.A., Stainier D.Y.R. (2017). Genetic compensation: A phenomenon in search of mechanisms. PLoS Genet..

[45] Burdakov D., Peleg-Raibstein D. (2020). The hypothalamus as a primary coordinator of memory updating. Physiol. Behav..

[46] Maroni M.J., Capri K.M., Arruda N.L., Gelineau R.R., Deane H.V., Concepcion H.A., DeCourcey H., Monteiro De Pina I.K., Cushman A.V., Chasse M.H. (2020). Substrain specific behavioral responses in male c57bl/6n and c57bl/6j mice to a shortened 21-h day and high-fat diet. Chronobiol. Int..

[47] Matsuo N., Takao K., Nakanishi K., Yamasaki N., Tanda K., Miyakawa T. (2010). Behavioral profiles of three c57bl/6 substrains. Front. Behav. Neurosci..

[48] Bryant C.D., Zhang N.N., Sokoloff G., Fanselow M.S., Ennes H.S., Palmer A.A., McRoberts J.A. (2008). Behavioral differences among c57bl/6 substrains: Implications for transgenic and knockout studies. J. Neurogenet..

[49] Gao A., Xia F., Guskjolen A.J., Ramsaran A.I., Santoro A., Josselyn S.A., Frankland P.W. (2018). Elevation of hippocampal neurogenesis induces a temporally graded pattern of forgetting of contextual fear memories. J. Neurosci..

[50] Hobson S.A., Holmes F.E., Kerr N.C., Pope R.J., Wynick D. (2006). Mice deficient for galanin receptor 2 have decreased neurite outgrowth from adult sensory neurons and impaired pain-like behaviour. J. Neurochem..

[51] Rotheneichner P., Romanelli P., Bieler L., Pagitsch S., Zaunmair P., Kreutzer C., Konig R., Marschallinger J., Aigner L., Couillard-Despres S. (2017). Tamoxifen activation of cre-recombinase has no persisting effects on adult neurogenesis or learning and anxiety. Front. Neurosci..

[52] Couillard-Despres S., Winner B., Schaubeck S., Aigner R., Vroemen M., Weidner N., Bogdahn U., Winkler J., Kuhn H.G., Aigner L. (2005). Doublecortin expression levels in adult brain reflect neurogenesis. Eur. J. Neurosci..

[53] Oberbauer E., Urmann C., Steffenhagen C., Bieler L., Brunner D., Furtner T., Humpel C., Baumer B., Bandtlow C., Couillard-Despres S. (2013). Chroman-like cyclic prenylflavonoids promote neuronal differentiation and neurite outgrowth and are neuroprotective. J. Nutr. Biochem..

[54] Locker F., Vidali S., Holub B.S., Stockinger J., Brunner S.M., Ebner S., Koller A., Trost A., Reitsamer H.A., Schwarzenbacher D. (2018). Lack of galanin receptor 3 alleviates psoriasis by altering vascularization, immune cell infiltration, and cytokine expression. J. Investig. Dermatol..

